# Removal of Acidic Organic Ionic Dyes from Water by Electrospinning a Polyacrylonitrile Composite MIL101(Fe)-NH_2_ Nanofiber Membrane

**DOI:** 10.3390/molecules27062035

**Published:** 2022-03-21

**Authors:** Jiao Jia, Hao Wu, Le Xu, Fengchun Dong, Yongtang Jia, Xi Liu

**Affiliations:** Guangdong-Hong Kong Joint Laboratory for New Textile Materials, Guangdong Functional Fiber and Textile Engineering Technology Research Center, School of Textile Materials and Engineering, Wuyi University, Jiangmen 529020, China; 17863625375@163.com (J.J.); wuhao_20211221@163.com (H.W.); xlxule@163.com (L.X.); dfchun@163.com (F.D.)

**Keywords:** electrospinning, MIL101(Fe)-NH_2_, functional nanofibers, water treatment membrane

## Abstract

A nanofiber metal–organic framework filter, a polyacrylonitrile (PAN) nanofiber membrane composite with an iron/2-amino-terephthalic acid-based metal–organic framework (MIL101(Fe)-NH_2_), was prepared by one-step electrospinning. MIL101(Fe)-NH_2_ was combined into the polymer nanofibers in situ. PAN-MIL101(Fe)-NH_2_ composite nanofiber membranes (NFMs) were prepared from a homogeneous spinning stock containing MIL101(Fe)-NH_2_ prebody fluid and PAN. Crystallization of MIL101(Fe)-NH_2_ and solidification of the polymer occurred simultaneously during electrospinning. The PAN-MIL101(Fe)-NH_2_ composite NFM showed that MIL101(Fe)-NH_2_ was uniformly distributed throughout the nanofiber and was used to adsorb and separate acidic organic ionic dyes from the aqueous solution. The results of Fourier transform infrared spectroscopy, energy-dispersive X-ray spectroscopy, and X-ray diffraction analysis showed that MIL101(Fe)-NH_2_ crystals were effectively bonded in the PAN nanofiber matrix, and the crystallinity of MIL101(Fe)-NH_2_ crystals remained good, while the distribution was uniform. Owing to the synergistic effect of PAN and the MIL101(Fe)-NH_2_ crystal, the PAN-MIL101(Fe)-NH_2_ composite NFM showed a fast adsorption rate for acidic ionic dyes. This study provides a reference for the rapid separation and purification of organic ionic dyes from wastewater.

## 1. Introduction

Environmental pollution caused by the discharge of wastewater containing organic dyes into water bodies is a worldwide problem that seriously endangers human health [1,2,3]. Organic dyes are used in various manufacturing industries, such as plastics, printing, textiles, and paper [4]. Failure to remove dyes from these wastes can contaminate water bodies, and organic dyes released into the water pose a major threat to the environment and human health because of their toxicity and carcinogenicity [5]. In addition, most dyes are very stable to light and oxidation, and degrading them is challenging [6,7]. To provide a solution, physical, chemical, and biological methods have been developed to treat organic dye contaminants [8,9,10]. Among these technologies, adsorption is extensively used because it is efficient, economically feasible, and simple to operate. Many materials, such as activated carbon, zeolite, ion exchange resins, and porous organic polymers, have been reported as adsorbents for the removal of organic dyes from water [11,12,13,14,15,16]. However, these adsorbents do not effectively separate the target dye for reuse. Considering the economic feasibility, developing new, efficient, and economical adsorbents for the removal of organic dyes from sewage is essential.

Metal–organic frameworks (MOFs) are a new type of inorganic–organic hybrid material whose inorganic metal ion clusters are connected to organic linkers via coordination bonds. Owing to their ease of preparation, adjustable pore size, strong modification ability, and large specific surface area, MOFs have been used as adsorbents to remove a variety of wastewater pollutants, including heavy metals, organic dyes, radionuclides, antibiotics, and inorganic ions [17,18]. Jhung et al. introduced urea or melamine on a porous MOF (MIL-101(Fe)) [19]. MIL-101(Fe) showed good adsorption capacities for saccharin, acesulfame, and cyclamate. Bu et al. reported a series of cationic MOFs and applied them to the selective separation of anionic dye molecules [20]. Huang et al. prepared an MOF (NH_2_-MIL-125) nanofiber hybrid film with photocatalytic performance and found that the membrane had a large adsorption capacity for methylene blue and sodium fluorescein, and the adsorption was mainly controlled by the steric hindrance of dye molecules [21]. Most importantly, the MOF membrane can be easily separated from the dye solution and reused through a visible light catalytic degradation process. However, most research conducts the preparation of MOFs in powder form, with complex recyclability, weak operation process, and potential secondary pollution shortcomings, which limits their practical application in wastewater treatment. From the perspective of the separation and recovery of adsorbents in wastewater, adsorption filtration membranes are more dominant than powder adsorbents, because separation membranes can offer better recovery.

Electrospinning is an effective and simple technique for the preparation of functionally advanced nanofiber membranes (NFMs) with a large specific surface area, high porosity, fine diameter, and modifiability [22,23,24]. Further, electrospun nanofibers have a larger specific surface area than thin films [25]. In recent years, the preparation of porous composite nanofibers by co-electrospinning has received considerable attention. These polymer and MOF composite NFMs are used in various fields, in particular for adsorption and separation [26,27,28,29]. Wang et al. designed a series of multifunctional porous membrane filters by processing porous membranes into nanoporous membranes [30]. These MOF filters can efficiently capture particles in real air pollution environments. Li et al. prepared a nanofiber MOF filter by electrostatic spinning technology for the adsorption and selective separation of cationic dyes in an aqueous solution [31]. Rana et al. used NFMs embedded with MOF-808 to adsorb heavy metal ions (Cd^2+^, Zn^2+^, Pb^2+^, and Hg^2+^) [32,33,34]. Kang et al. reported a novel metal–organic framework membrane adsorbent for water treatment, in which two MOFs (ZIF-8 and ZIF-L) were grown in porous A-alumina carriers to form membranes for the adsorption of dye molecules, and they found that the water permeability of ZIF-L-based membrane adsorbents far exceeded that of most existing membrane adsorbents [35]. Zhao et al., with the help of polyvinylpyrrolidone, constructed interlinked mesopores in electrospun zeolite imidazole-skeleton-8(ZIF-8)/PAN fibers, exposing more adsorption sites for ZIF-8 and enabling ZIF-8 to be more stable. In addition, the mesopores enhanced the diffusion of pollutant molecules and created an MOF polymer interface in the fiber, which improved the adsorption rate and adsorption capacity, respectively. The fibers were used to adsorb tetracycline, an antibiotic, from water [36]. Maya et al. demonstrated a general scheme for fabricating freestanding polymer fibers embedded in porous fibers, wherein the fibers themselves served as microreactors for the in situ growth of porous fiber crystals. The fibers embedded with MOFs were obtained using a two-step method [37].

In this study, we designed and prepared an iron/2-amino-terephthalic acid-based metal–organic framework (MIL101(Fe)-NH_2_) powder and propose a simple one-step electrospinning strategy for the in situ binding of MIL101(Fe)-NH_2_ to polymer nanofibers. PAN-MIL101(Fe)-NH_2_ composite NFMs were prepared from a homogeneous stock solution containing MIL101(Fe)-NH_2_ prebody fluid and PAN. The crystallization of MOFs and solidification of polymers occurred simultaneously during electrospinning, thus avoiding the aggregation and complex multi-step manufacturing challenges of conventional methods. The PAN-MIL101(Fe)-NH_2_ composite NFM showed that the MOF was uniformly distributed throughout the nanofiber. The composite nanofiber membrane was used to adsorb and separate organic ionic dyes from an aqueous solution. The results of Fourier transform infrared spectroscopy, (FT-IR), energy-dispersive X-ray spectroscopy (EDS), and X-ray diffraction analysis (XRD) showed that MIL101(Fe)-NH_2_ crystals were effectively distributed on the PAN nanofiber matrix, with good crystallinity. This study provides a new perspective for electrospinning MOF NFMs as filters for the rapid separation and purification of organic dyes in practical wastewater treatment.

## 2. Materials and Methods

### 2.1. Materials

PAN (M_w_ = 150 kDa) was purchased from Shunjie Plastic Technology Co., Ltd. (Nanjing, China). FeCl_3_·6H_2_O was purchased from Tianjin Best Chemical Co., Ltd. (Tianjin, China). 2-amino-terephthalic acid, rhodamine B, methyl orange, and Congo red were purchased from Anergy Chemical; indigo carmine was purchased from OKA; methylene blue trihydrate, acid red 27, and acid blue 93 were purchased from Aladdin Chemical. Chlorophyll was purchased from NJDULY in Nanjing. N,N-dimethylformamide (DMF) was purchased from Tianjin Best Chemical. All chemicals were of analytical grade and used without further purification.

### 2.2. Methods

#### 2.2.1. Preparation of MIL101(Fe)-NH_2_ Powder

MIL101(Fe)-NH_2_ powder was synthesized according to a previously reported method [38]. FeCl_3_·6H_2_O, 2-amino-terephthalic acid and N-N dimethylformamide were added to a 100 mL PTFE-lined autoclave. The autoclave was covered and kept at 130 °C for 24 h to obtain a yellow crystal. The synthesized MOF crystals were refluxed in DMF and ethanol in a Soxhlet extractor and then dried under vacuum at 90 °C for 24 h to obtain MIL101(Fe)-NH_2_ powder.

#### 2.2.2. Preparation of PAN and PAN-MIL101(Fe)-NH_2_ Composite NFMs

The PAN nanofiber membrane was prepared by electrospinning the PAN spinning solution (1000 mg PAN in 9 mL DMF) at a voltage of 20 kV, a flow rate of 0.5 mL/h, and a receiving distance of 20 cm. The obtained PAN nanofiber membrane was washed with methanol and dried in a vacuum oven at 80 °C for 24 h.

The PAN-MIL101(Fe)-NH_2_ composite nanofiber membrane was prepared by one-step electrospinning. First, Liquid A was prepared by dissolving 500 mg FeCl_3_·6H_2_O and 250 mg 2-amino-terephthalic acid in 4.25 mL DMF, and Liquid B was prepared by dissolving 900 mg PAN and 100 mg 2-amino-terephthalic acid in 9 mL DMF. Then, Liquid A and Liquid B were mixed and stirred at 60 °C for 3 h to obtain a homogenized spinning solution. The electrospinning process was carried out at a flow rate of 0.5 mL/h, a voltage of 20 kV, and a receiving distance of 20 cm. Finally, the obtained PAN-MIL101(Fe)-NH_2_ composite nanofiber membrane was washed with methanol and dried in a vacuum oven at 80 °C for 24 h.

### 2.3. Characterization

ZEISS Sigma500 was used to capture the surface morphology and elemental distribution of the NFMs. A Fourier transform infrared spectrometer (Nicolet IS 10) was used to record the FT-IR data. The crystallinity of the synthesized MIL101(Fe)-NH_2_ powder and PAN-MIL101(Fe)-NH_2_ composite nanofiber films was determined using an X-ray diffractometer. X-ray photoelectron spectroscopy (XPS) was performed using a Thermo Scientific ESCALAB spectrometer (Thermo Fly) at 40 kV. The spectra of the organic dye solutions were recorded using a UV-visible spectrophotometer (Shimadzu UV-3600) in the wavelength range of 200–800 nm. 

### 2.4. Adsorption Experiment

The filtration and adsorption experiments of the PAN nanofiber membrane and PAN-MIL101(Fe)-NH_2_ composite nanofiber membrane were carried out in the continuous filtration system established in the laboratory (Figure S1). An effective filtration area of 3 cm^2^ (approximately 30 mg) and dye solution were placed on both sides of the peristaltic pump. An organic ionic dye solution with an initial concentration of 10 mg/L was configured and forced through a peristaltic pump (China BT300L) at a constant flow rate (flow rate 1 mL/min). The concentration of the dye solution was determined using UV-Vis spectrophotometry.

The adsorption experiment of the MIL101(Fe)-NH_2_ powder was also carried out by a peristaltic pump. A thin layer of MIL101(Fe)-NH_2_ powder (about 30 mg) was spread between two layers of filter paper, and the initial concentration of organic ionic dye solution was 10 mg/L, forced through the peristaltic pump (China BT300L) at a constant flow rate (flow rate is 1 mL/min). The concentration of dye solution was determined by UV-visible spectrophotometry.

## 3. Results and Discussion

### 3.1. Preparation and Properties of the PAN-MIL101(Fe)-NH_2_ Composite NFM

**Scheme 1:**
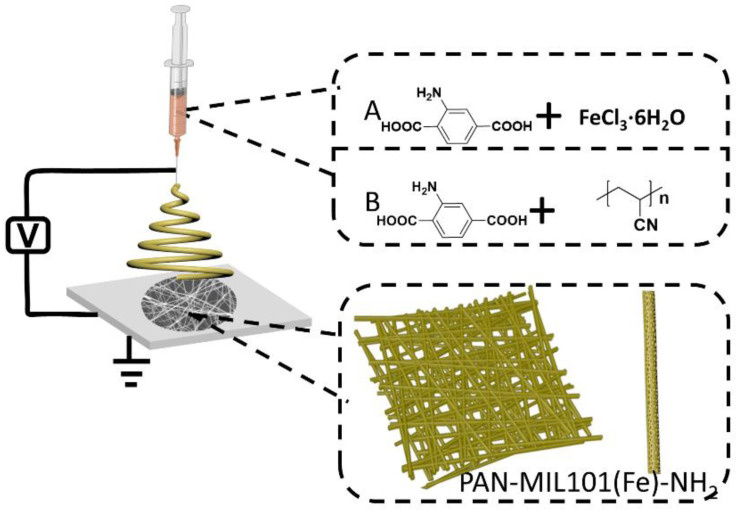
Schematic illustration of the preparation of the PAN-MIL-100(Fe)-NH_2_ NFM.

**Figure 1:**
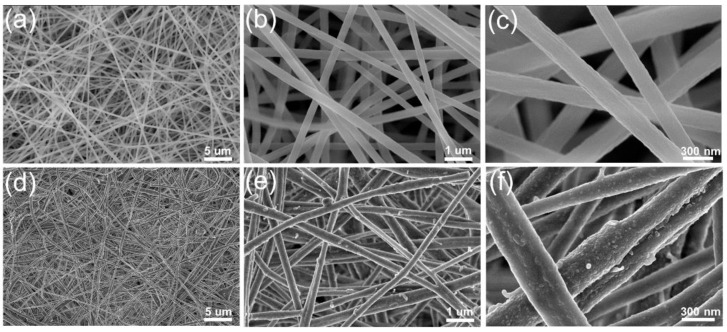
Scanning electron microscopy (SEM) images of the PAN NFM (**a**–**c**) and PAN-MIL101(Fe)-NH_2_ NFM (**d**–**f**).

Previous studies have shown that the reaction site of the corresponding organic acid ligands in spinning solution leads to the growth of MOFs in porous materials [39,40]. For the porous coordination polymer MIL101(Fe)-NH_2_, iron ions (inorganic metal ions, Fe^3+^), and 2-amino-terephthalic acid (organic ligands) can be used as the reaction sites. Scheme 1 illustrates the process of preparing the PAN-MIL101(Fe)-NH_2_ composite NFM. The crystallization of MIL101(Fe)-NH_2_ and the solidification of the polymer occurred simultaneously during the electrostatic spinning process. In consideration of its spinnability, 2-amino-terephthalic acid was added to the PAN spinning solution as a spinning solution supporting MIL101(Fe)-NH_2_, in which PAN acted as the polymer skeleton. Further, 2-amino-terephthalic acid acted as the initial reaction site for MIL101(Fe)-NH_2_ growth. The morphologies of the two membranes were tested using scanning electron microscopy (SEM). As shown in Figure 1, the nanofiber morphologies were formed in the PAN and PAN-MIL101(Fe)-NH_2_ NFM. The two NFMs showed similar porous nanofibrous network structures with approximate diameters of ≈200 nm. However, compared to the smooth surfaces of the single PAN fibers, the PAN-MIL101(Fe)-NH_2_ fibers showed a rough surface morphology, which may be attributed to the presentation of the MIL101(Fe)-NH_2_ MOF nanoparticles. Overall, the morphology study indicated that the PAN composite MIL101(Fe)-NH_2_ NFM was successfully obtained. 

**Figure 2:**
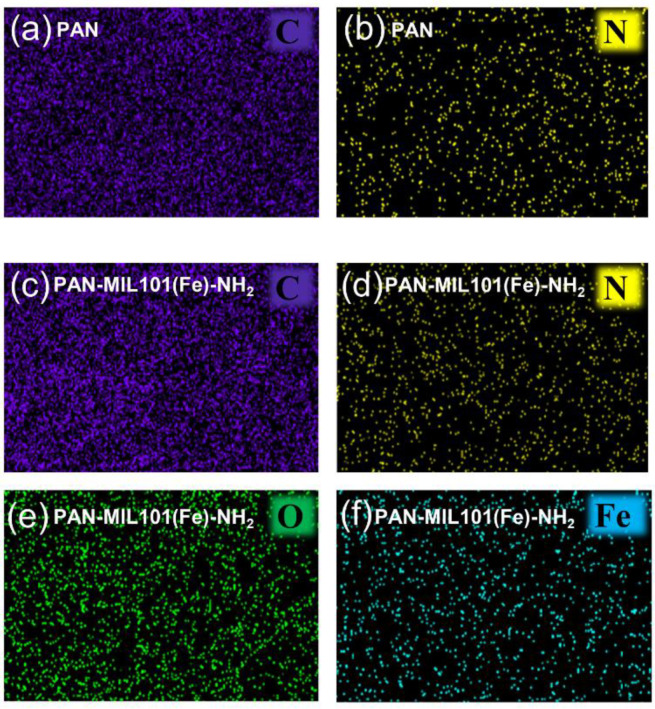
EDS mapping images of the PAN NFM: (**a**) C and (**b**) N; and EDS mapping images of the PAN-MIL101(Fe)-NH_2_ NFM: (**c**) C, (**d**) N, (**e**) N, and (**f**) Fe.

To further analyze the elemental composition and distribution, the corresponding EDS mapping experiments of the PAN and PAN-MIL101(Fe)-NH_2_ NFM were performed. As shown in Figure 2a,b, the PAN NFM showed uniform distributions of the C and N elements. Figure 2c–f shows the existence of the C, N, O, and Fe elements in the PAN-MIL101(Fe)-NH_2_ composite NFM. Specifically, the Fe element showed a uniform distribution in the PAN-MIL101(Fe)-NH_2_ NFM. Thus, the EDS results preliminarily proved the existence of MIL101(Fe)-NH_2_ in the PAN-MIL101(Fe)-NH_2_ nanofiber.

**Figure 3:**
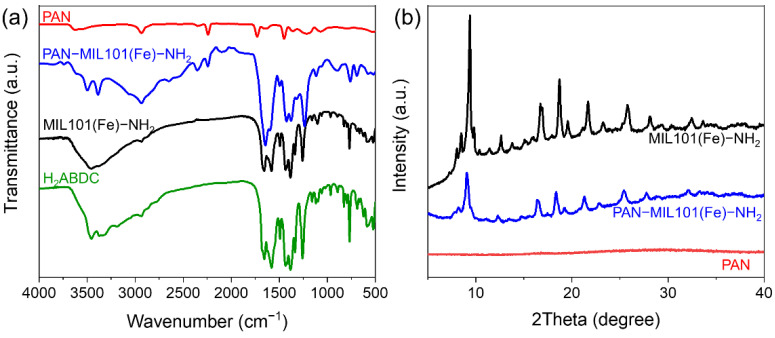
(**a**) FT-IR spectra of the PAN, PAN-MIL101(Fe)-NH_2_, MIL101(Fe)-NH_2_, and 2-amino-terephthalic acid. (**b**) XRD spectra of the PAN, PAN-MIL101(Fe)-NH_2_, and MIL101(Fe)-NH_2_.

Figure 3a shows the FT-IR spectra of the PAN, PAN-MIL101(Fe)-NH_2_, and MIL101(Fe)-NH_2_. The PAN NFM showed characteristic absorption peaks at 2243 cm^−1^ and 1738 cm^−1^, corresponding to the tensile vibration of C≡N and C=O bonds, respectively, which was due to the commercial PAN being usually contained with ~91 wt.% acrylonitrile monomer and ~9 wt.% methyl acrylate comonomer [31,41,42]. For MIL101(Fe)-NH_2_, the characteristic absorption peaks at 1450–1620 cm^−1^ and 660–780 cm^−1^ corresponded to the tensile vibration of the benzene ring and substituent group on the benzene ring, respectively, and the peak at 1660 cm^−1^ was attributed to the C=O tensile vibration of the carboxyl group. In the infrared spectrum of the PAN-MIL101(Fe)-NH_2_ composite NFM, all the corresponding key characteristics of the MIL101(Fe)-NH_2_ and PAN could be observed, which further proved that the MIL101(Fe)-NH_2_ crystals were successfully combined with the PAN nanofibers.

The XRD patterns of the PAN, PAN-MIL101(Fe)-NH_2_, and MIL101(Fe)-NH_2_ are compared in Figure 3b to determine the crystal structure. The crystallinity of the MIL101(Fe)-NH_2_ powder was consistent with the recorded data. For pure PAN NFM, there was no obvious peak corresponding to the amorphous form of PAN. The XRD diffraction pattern of the PAN-MIL101(Fe)-NH_2_ was very consistent with that of the original MIL101(Fe)-NH_2_, indicating that the growth of the MIL101(Fe)-NH_2_ powder was well realized during the preparation of the composite nanofibers.

**Figure 4:**
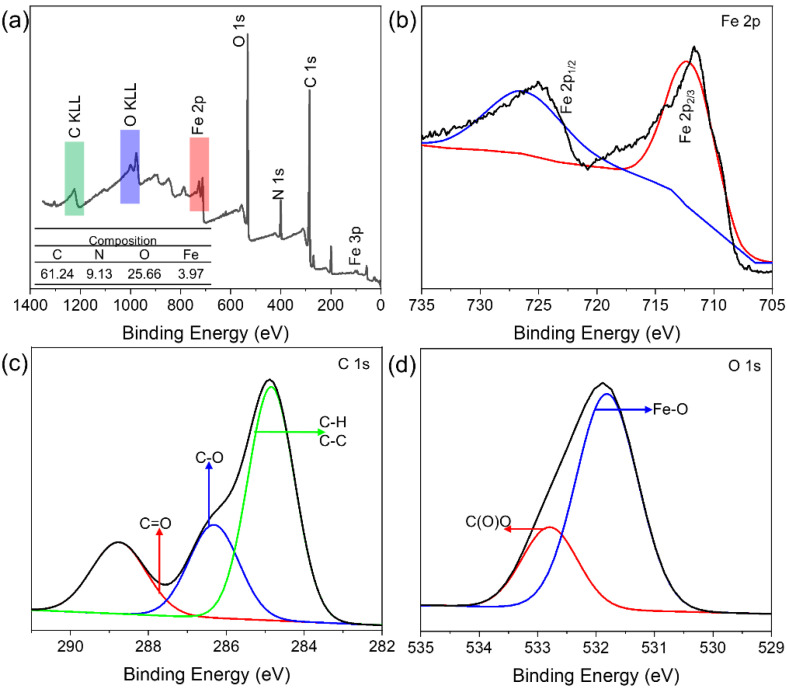
XPS spectra of the PAN-MIL101(Fe)-NH_2_: (**a**) broad spectrum, (**b**) Fe2p, (**c**) C1s, and (**d**) O1s spectra.

The elemental content of the composite PAN-MIL101(Fe)-NH_2_ NFM was examined using XPS, and the results are shown in Figure 4. Figure 4a preliminary exhibits the existence of the C, O, N, and Fe elements, which was consistent with the elements of PAN and MIL101(Fe)-NH_2_. Meanwhile, the contents of C, O, N, and Fe in the PAN-MIL101(Fe)-NH_2_ composite NFM were 61.24%, 9.13%, 25.66%, and 3.97%, respectively. Figure 4b shows a high-resolution map of Fe2p, where the binding energies of Fe2p_1/2_ and Fe2p_3/2_ were concentrated at 711.71 eV and 724.93 eV, respectively, proving that iron existed in a trivalent chemical state. Figure 4c shows a high-resolution atlas of C1s. The peaks at 288.65 eV, 286.37 eV, and 284.82 eV were due to the C=O, C–O, and C–H/C–C bonds, respectively. As shown in Figure 4d, the peak value at 532.82 eV was attributed to the oxygen in the carboxylic group of 2-amino-terephthalic acid, and that at 531.91 eV belonged to the oxygen in the Fe–O bond in MIL101(Fe)-NH_2_. 

### 3.2. Adsorption Effect of the PAN-MIL101(Fe)-NH_2_ Composite NFM

**Figure 5:**
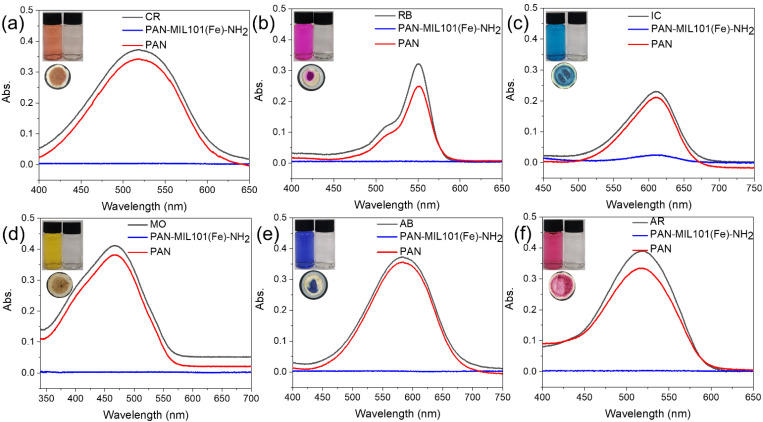
Absorption spectra of the six dyes by the PAN and PAN-MIL101(Fe)-NH_2_ NFM (**a**–**f**). Insets are the color contrast of the solution before and after the adsorption and the optical image of the PAN-MIL101(Fe)-NH_2_ NFM after adsorption.

The adsorption performance of the PAN-MIL101(Fe)-NH_2_ composite NFM filter was evaluated by separating the dyes from polluted water, and six acidic dye molecules (Congo red (CR), rhodamine B (RB), indigo carmine (IC), methyl orange (MO), acid blue 93 (AB), and acid red 27 (AR)) were selected as the experimental models. Figure 5 shows the adsorption capacities of the PAN and PAN-MIL101(Fe)-NH_2_ composite NFM for the six acidic ionic dyes. After filtration by PAN nanofibers, the absorption peaks of different ionic dyes were reduced to a certain extent, which proved that a single PAN NFM had a certain removal efficiency for acidic ionic dyes. As illustrated in Figure 5a–f, after filtration by the PAN-MIL101(Fe)-NH_2_ composite NFM, the absorption peaks of different ionic dyes showed obvious changes, and the dye solution filtered by the PAN-MIL101(Fe)-NH_2_ presented a transparent color. The color of the PAN-MIL101(Fe)-NH_2_ nanofibers changed from light yellow to a color similar to that of the dye. The removal efficiencies of the PAN and PAN-MIL101(Fe)-NH_2_ NFM for the six dyes were calculated based on the following formula:

$$Removal\left(\%\right)=\frac{\left(A_{0}-A_{t}\right)}{A_{0}}\times 100\%$$

which *A*_0_ is the absorption value at the absorption peak of the initial sample and *A_t_* is the corresponding absorption value after each filtration. As shown in Table S1, the removal efficiencies of the PAN NFM for CR, RB, IC, MO, AB, and AR were 8.01%, 21.13%, 8.21%, 7.28%, 4.47%, and 17.31%, respectively. The PAN-MIL101(Fe)-NH_2_ NFM showed a highly efficient adsorption capacity, which exhibited removal efficiencies of for CR, RB, IC, MO, AB, and AR of 98.76%, 96.03%, 92.35%, 99.12%, 98.02%, and 99.52%, respectively. The results, combined with the related removal efficiencies of MIL101(Fe)-NH_2_ powder (Figure S2 and Table S1), showed that the PAN-MIL101(Fe)-NH_2_ composite NFM could successfully filter acidic ionic dyes from aqueous solutions. 

### 3.3. Adsorption Kinetics of the PAN-MIL101(Fe)-NH_2_ Composite NFM

**Figure 6:**
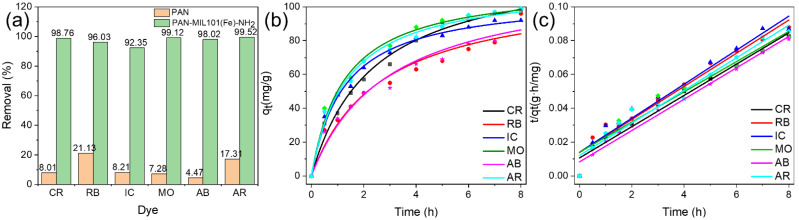
(**a**) Removal efficiency of dyes by the PAN and PAN-MIL101(Fe)-NH_2_ NFM. (**b**) Adsorption kinetics and (**c**) pseudo-second-order kinetics of the PAN-MIL101(Fe)-NH_2_ for Congo red, rhodamine B, indigo carmine, methyl orange, acid blue 93, and acid red 27.

The time-varying experimental results shown in Figure 6 showed that the PAN-MIL101(Fe)-NH_2_ composite NFM exhibited rapid adsorption of different dyes, and the adsorption process reached equilibrium at 8 h, under adsorption conditions (C_0_: 10 mg/L, adsorbent dosage: 30 mg/mL). Figure 6b further analyzes the experimental data changing with time using the pseudo-second-order dynamics model, and its linear Equation (1) is expressed as follows:

(1) $$\frac{t}{q_{t}}=\frac{1}{k_{2}q_{e}{}^{2}}+\frac{t}{q_{e}}$$

where *q_t_* and *q_e_* (mg/g) are *t* and the adsorption capacity at equilibrium, respectively. *k*_2_ is the pseudo-second-order model rate constant [43]. The linear curves are shown in Figure 6c.

### 3.4. Adsorption Isotherms of the PAN-MIL101(Fe)-NH_2_ Composite NFMs

**Figure 7:**
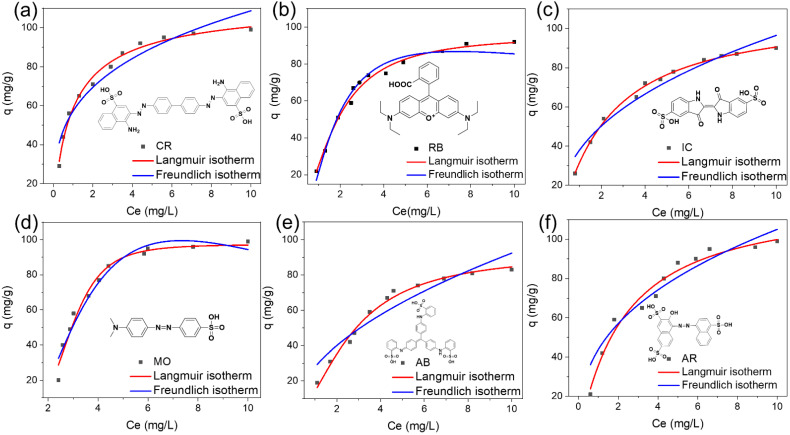
Adsorption isotherms for Congo red (**a**), rhodamine B (**b**), indigo carmine (**c**), methyl orange (**d**), acid blue 93 (**e**), and acid red 27 (**f**).

To understand the maximum adsorption capacity and the interaction between the adsorbent and adsorbent, the adsorption isotherms of different dyes on the PAN-MIL101(Fe)-NH_2_ composite NFMs were obtained. Two widely used isotherm models (Langmuir and Freundlich) were used to analyze the isotherm data (Figure 7). The linear equation is as follows:

Langmuir isotherms (homogeneous and monolayer adsorption) (Equation (2)) [44]:

(2) $$\frac{C_{e}}{q_{e}}=\frac{C_{e}}{q_{m}}+\frac{1}{bq_{m}}$$

Freundlich isotherms (heterogeneous and multilayer adsorption) (Equation (3)) [44]:

(3) $$\log q_{e}=\log K_{F}+\frac{1}{n}\log C_{e}$$

where *q_e_* (mg/g) is the equilibrium adsorption capacity, *C_e_* (mg/L) is the equilibrium concentration, and *q_m_* and *b* are Langmuir constants, which are related to the maximum adsorption capacity and binding energy, respectively. *K_F_* and *n* represent the empirical constants of the Freundlich constant and the heterogeneity factor, respectively. The results show that the Langmuir isotherm model could describe the two pollutants well, indicating that monolayer adsorption and chemisorption may exist. 

### 3.5. Maximum Load of the PAN-MIL101(Fe)-NH_2_ Composite NFM

**Figure 8:**
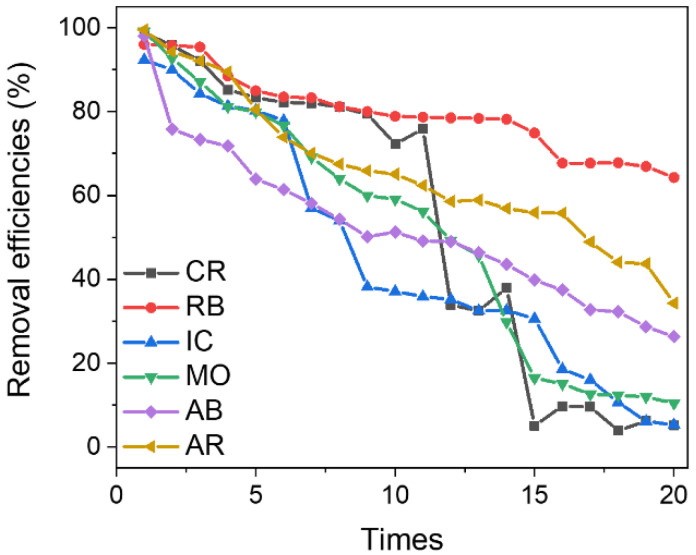
Long-term removal efficiencies of the PAN-MIL101(Fe)-NH_2_ NFM for Congo red (CR), rhodamine B (RB), indigo carmine (IC), methyl orange (MO), acid blue 93 (AB), and acid red 27 (AR).

Figure 8 shows the long-term removal efficiencies of the PAN-MIL10 (Fe)-NH_2_ NFM for different dyes under cycling. The PAN-MIL10 (Fe)-NH_2_ NFM showed high removal efficiencies of >70% for Congo red and rhodamine B after 12 cycles. According to the long-term absorption results, we then calculated the maximum unit load of the PAN-MIL10 (Fe)-NH_2_ NFM for different dyes under cycling. As shown in Figure S3 and Table S2, the maximum unit load of the PAN-MIL10 (Fe)-NH_2_ NFM for Congo red could reach 200 mg/g. When the maximum loading was reached, the removal effect of the PAN-MIL101(Fe)-NH_2_ composite nanofiber membrane decreased significantly. The maximum unit load of rhodamine B dye per gram of the PAN-MIL101(Fe)-NH_2_ composite NFM was 333 mg/g. After reaching the maximum loading capacity, the dye removal efficiency of the PAN-MIL101(Fe)-NH_2_ composite NFM decreased significantly. Moreover, the XRD results (Figure S4) of PAN-MIL101(Fe)-NH_2_ NFM before and after dye adsorption showed its stability under long-term cycling. The results showed that the PAN-MIL101(Fe)-NH_2_ composite NFM had a good adsorption removal effect and high adsorption capacity for acidic ionic dyes. 

## 4. Conclusions

In conclusion, we successfully prepared an MIL101(Fe)-NH_2_-cured electrospun PAN film (PAN-MIL101(Fe)-NH_2_) via electrostatic spinning. Further, 2-amino-terephthalic acid is essential for the growth of MIL101(Fe)-NH_2_ in prodromal fluids. The PAN-MIL101(Fe)-NH_2_ composite NFM had a good adsorption capacity for organic ionic dyes, and it could achieve high filtration performance. The adsorption kinetics of the PAN-MIL101(Fe)-NH_2_ composite NFMs on acid organic ionic dyes showed that its adsorption on acid organic ionic dyes was more in line with quasi-second-order kinetics, indicating that the adsorption rate was mainly controlled by the chemical adsorption mechanism. Furthermore, the isothermal model of the PAN-MIL101(Fe)-NH_2_ composite NFM for acidic organic ionic dyes was more consistent with the Langmuir isothermal model. The results showed that PAN-MIL101(Fe)-NH_2_ composite NFMs are a highly competitive candidate material for wastewater treatment. We believe that our study will facilitate research on multifunctional MOF-based materials.

## Data Availability

Not applicable.

## Supplementary Materials

The following are available online at https://www.mdpi.com/article/10.3390/molecules27062035/s1, Figure S1: Schematic diagram of the adsorption device, Figure S2: Removal effect of the MIL101(Fe)-NH_2_ powder for the dyes, Figure S3: Long-term removal efficiencies of the PAN-MIL101(Fe)-NH_2_ for the dyes, Figure S4: XRD spectra of the PAN-MIL101(Fe)-NH_2_ adsorption of dye comparison before and after, Table S1: Removal efficiencies of the dyes by the PAN, PAN-MIL101(Fe)-NH_2_ NFMs, and MIL101(Fe)-NH_2_ powder, Table S2: Maximum loading weights of the PAN-MIL101(Fe)-NH_2_ NFM for the dyes.

## Sample Availability

Samples of PAN and PAN-MIL101(Fe)-NH_2_ NFMs are available from the authors with appropriate requests.
