# Supplementary material for: Removal of Acidic Organic Ionic Dyes from Water by Electrospinning a Polyacrylonitrile Composite MIL101(Fe)-NH2 Nanofiber Membrane
Source: Molecules. 2022 Mar 21;27(6):2035. doi: 10.3390/molecules27062035 (PMC8954605; doi:10.3390/molecules27062035)
Supplement: Supplementary file 1 [file molecules-27-02035-s001.zip › molecules-1631000-supplementary.pdf]

molecules

Supplementary Materials

Removal of acidic organic ionic dyes from water by electrospinning a polyacrylonitrile composite MIL101(Fe)-NH_2 nanofiber membrane

Jiao Jia, Hao Wu, Le Xu, Fengchun Dong, Yongtang Jia* and Xi Liu*

Guangdong-Hong Kong Joint Laboratory for New Textile Materials, Guangdong Functional Fiber and Textile Engineering Technology Research Center, School of Textile Materials and Engineering, Wuyi University, Jiangmen 529020, China
*Correspondence: yongtjh@163.com; liuxi_wyu@163.com or liuxi@wyu.edu.cn

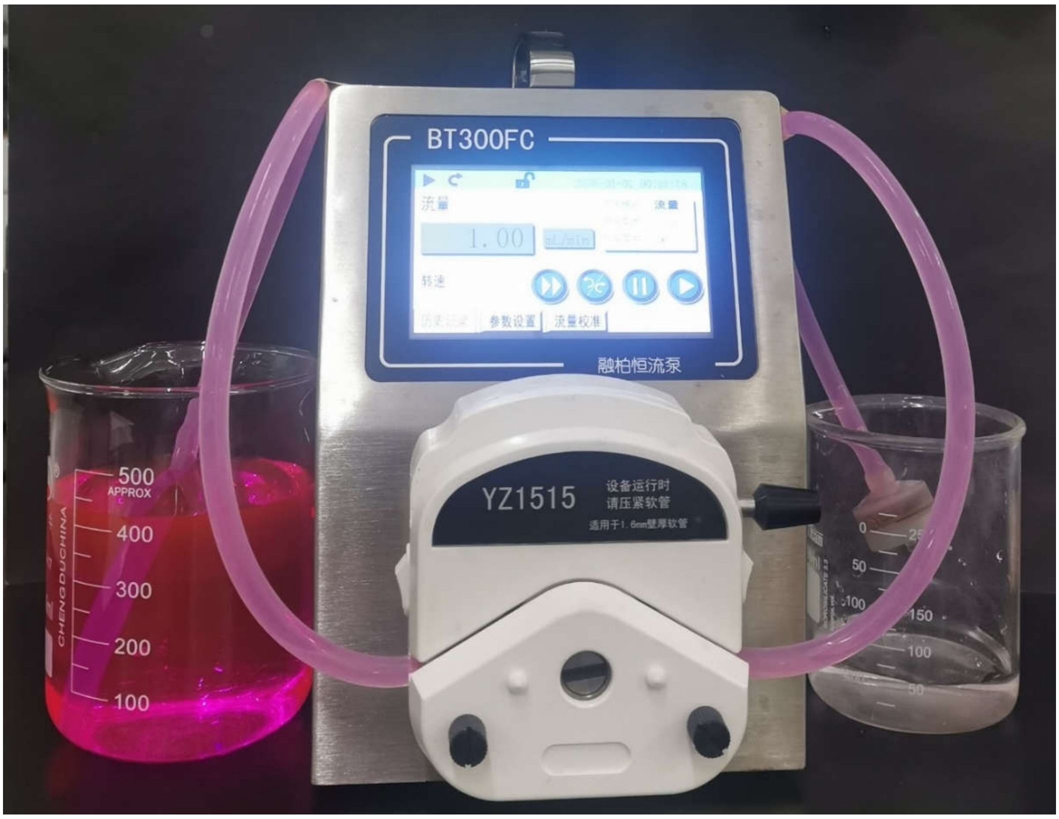

Figure S1: Schematic diagram of adsorption device.

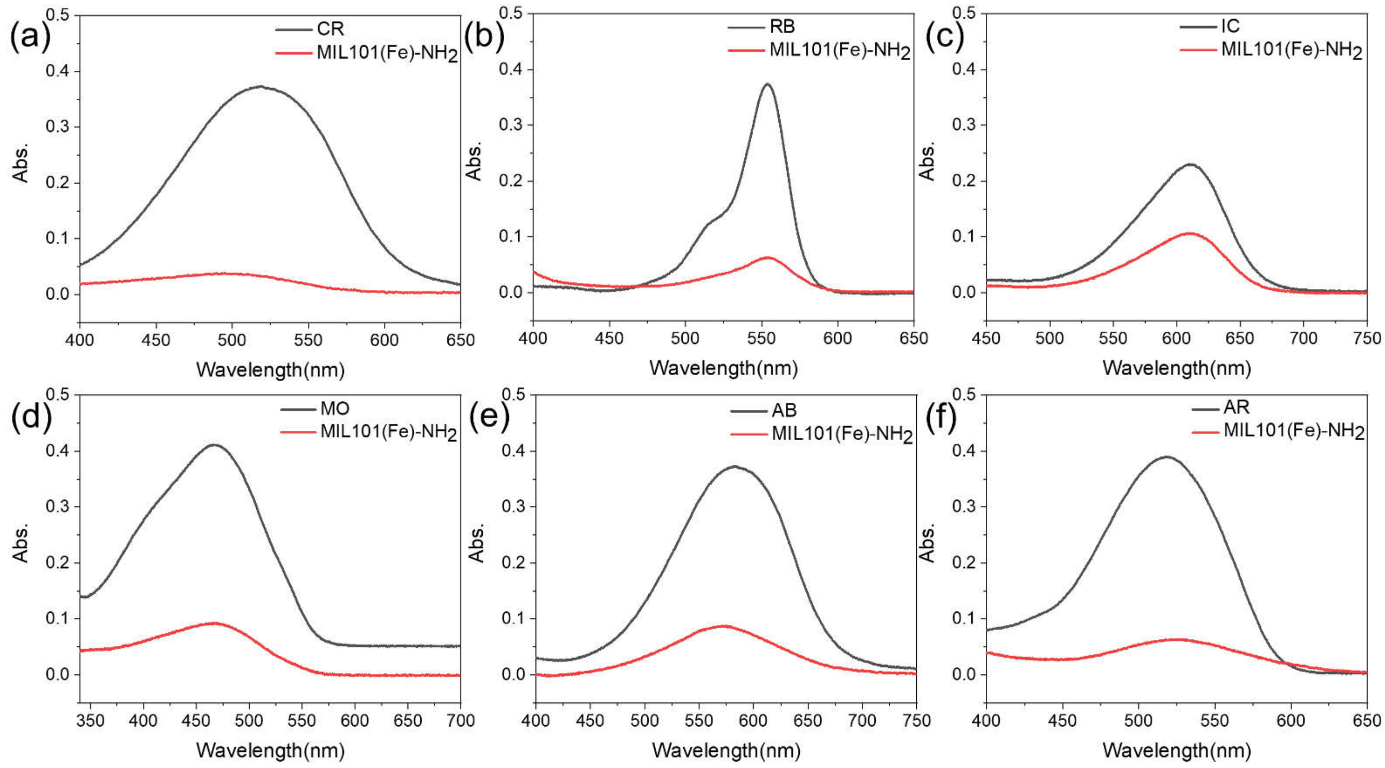

Figure S2: Removal effect of MIL101(Fe)-NH_2 powder for congo red(a), rhodamine B(b), indigo carmine(c), methyl orange(d), acid blue 93(e) and acid red 27(f).

The removal efficiencies of MIL101(Fe)-NH2 powder on congo red, rhodamine B, indigo carmine, methyl orange, acid blue 93 and acid red 27 were 91.36%, 83.21%, 53.98, 77.51%, 76.73% and 98.41%, respectively. However, after combining MIL101(Fe)-NH_2 powder with PAN, Due to the synergistic effect of MIL101(Fe)-NH_2 powder and PAN, MIL101(Fe)-NH_2 powder was evenly distributed on the surface of PAN nanofibers, increasing the contact area with dye solution during the adsorption process, thus increasing the adsorption performance of dyes.

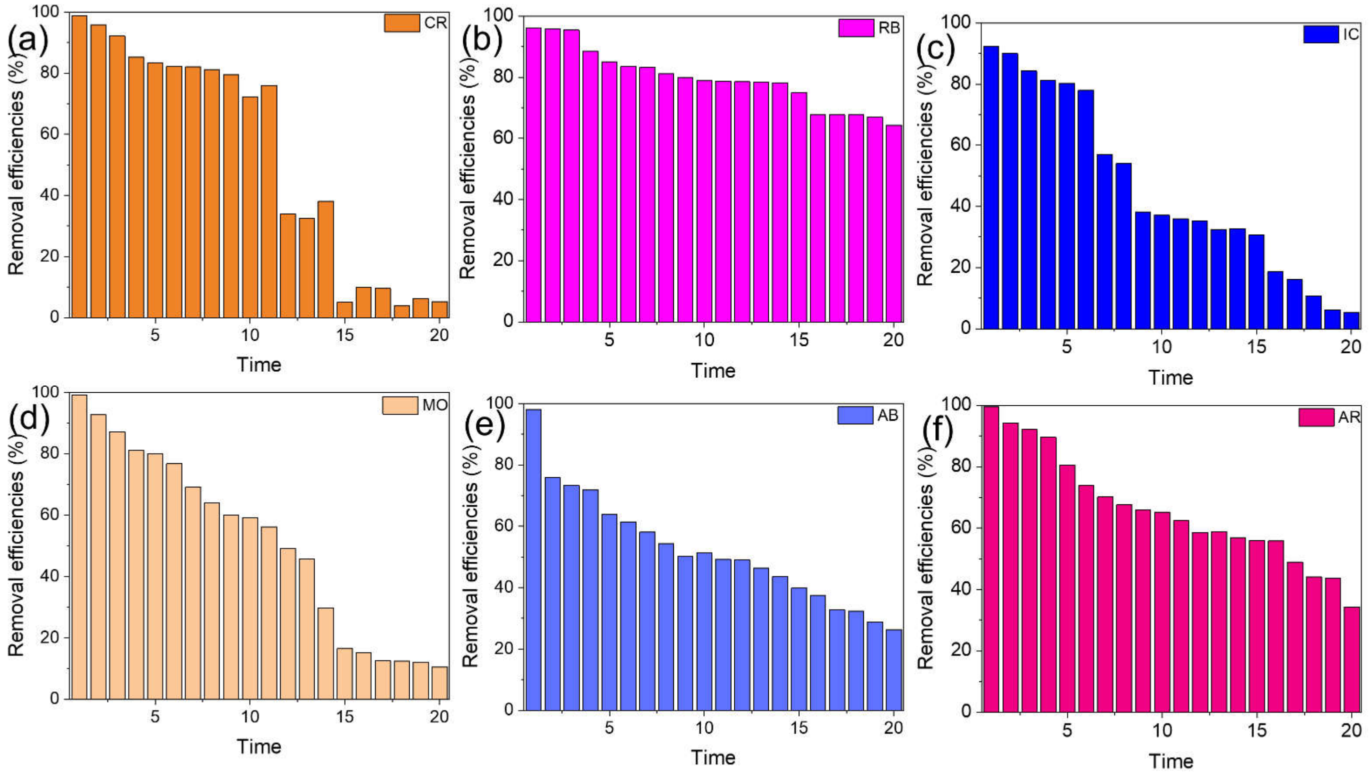

Figure S3: Long-term removal efficiencies of PAN-MIL101(Fe)-NH_2 for congo red(a), rhodamine B(b), indigo carmine(c), methyl orange(d), acid blue 93(e) and acid red 27(f).

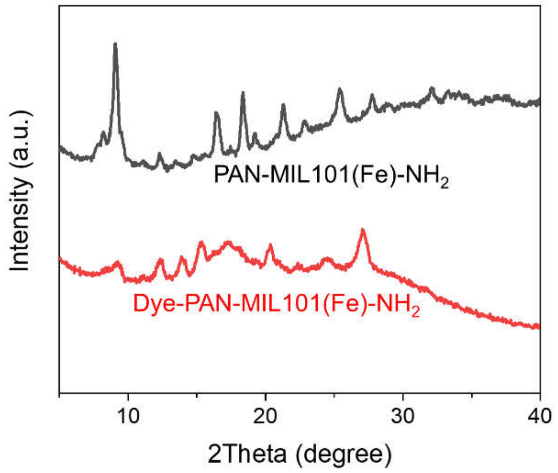

Figure S4: XRD spectra of PAN-MIL101(Fe)-NH_2 comparison before and after dye adsorption

XRD tests were carried out on PAN-MIL101(Fe)-NH_2 composite nanofiber membrane before and after dye adsorption. The test results showed that the crystallinity of PAN-MIL101(Fe)-NH_2 composite nanofiber membrane after dye adsorption decreased to a certain extent, which may be attributed to the large content of dye molecules in the NFM and the diffraction signals of MIL101(Fe)-NH_2 MOF interfered.

Table S1: Removal efficiencies of congo Red, rhodamine B, indigo carmine, methyl orange, acid blue 93 and acid red 27 by PAN,PAN-MIL101(Fe)-NH_2, and MIL101(Fe)-NH_2 powder.

	Dye name	A_t	A_0	Removal efficiency(%)
PAN	Congo Red (CR)	0.3483	0.3790	8.01
	Rhodamine B (RB)	0.2366	0.3001	21.13
	Indigo Carmine (IC)	0.2201	0.2399	8.24
	Methyl orange (MO)	0.3814	0.4114	7.28
	Acid Blue 93 (AB)	0.3567	0.3734	4.47
	Acid Red 27 (AR)	0.3471	0.4198	17.31
PAN-MIL101(Fe)-NH_2.	Congo Red (CR)	0.0047	0.3790	98.76
	Rhodamine B (RB)	0.0119	0.3001	96.03
	Indigo Carmine (IC)	0.0184	0.2399	92.35
	Methyl orange (MO)	0.0036	0.4114	99.12
	Acid Blue 93 (AB)	0.0074	0.3734	98.02
	Acid Red 27 (AR)	0.0020	0.4198	99.52
MIL101(Fe)-NH_2 powder	Congo Red (CR)	0.03209	0.3715	91.36
	Rhodamine B (RB)	0.0626	0.3729	83.21
	Indigo Carmine (IC)	0.1056	0.2295	53.98
	Methyl orange (MO)	0.0915	0.4069	77.51
	Acid Blue 93 (AB)	0.0844	0.3628	76.73
	Acid Red 27 (AR)	0.0062	0.3902	98.41

Table S2: Maximum loading weights of PAN-MIL101(Fe)-NH_2 on congo Red, rhodamine B, indigo carmine, methyl orange, acid blue 93 and acid red 27.

Dye	PAN-MIL101(Fe)-NH_2 (g)	C_e (mg/L)	Cycling Times	Maximum loading (mg/g)
Congo Red (CR)	0.038	7.587	12	199.66
Rhodamine B (RB)	0.024	8	12	333.33
Indigo Carmine (IC)	0.031	7.79	6	259.66
Methyl orange (MO)	0.028	5.617	11	200.61
Acid Blue 93 (AB)	0.026	5.19	10	199.62
Acid Red 27 (AR)	0.024	6.51	10	271.25
